# Analyzing public sentiment toward GMOs via social media between 2019-2021

**DOI:** 10.1080/21645698.2023.2190294

**Published:** 2023-03-22

**Authors:** Manreet Sohi, Maurice Pitesky, Joseph Gendreau

**Affiliations:** a Department of Computer Science, School of Letters and Science, University of California, Davis, CA, USA; b Department of Population Health and Reproduction, School of Veterinary Medicine-Cooperative Extension, University of California, Davis, CA, USA

**Keywords:** Gmos, sentiment analysis, social media, web crawling

## Abstract

Genetically modified organisms or GMOs offer significant advantages in food production, including increased yield, decreased pesticide usage, and better disease resistance. However, adoption and public sentiment toward GMOs is highly variable. Without positive sentiment toward GMOs, consumption of GMO-based foods may not have an adequate market for further investment. In order to better understand overall public sentiment toward GMO-based foods, a Boolean search was created using a commercial web-crawling service to collect and analyze public sentiment of GMOs across multiple social media and web-based services from May 1, 2019, to May 31, 2021. The Boolean query identified 2 million mentions of GMOs during the study period. Using the commercial software’s sentiment analysis (i.e. classifying mentions as either neutral, negative, or positive), 54% of the mentions were categorized as having a neutral sentiment, 32% as having a negative sentiment, and 14% as having a positive sentiment. Further emotional analysis (classifying posts by the emotion expressed, e.g., disgust, joy, sadness, anger, fear, surprise) produced by the software shows that the majority of the mentions were categorized as expressing a negative emotion: 31% of mentions expressed disgust, 28% joy, 18% sadness, 16% anger, 7% fear, and 1% surprise. Among the various social media sources collected, Twitter was the main source of data, providing 62% of the total 2 million mentions, followed by 14% from news sources and 12% from Reddit. These types of data can be used to better understand trends in sentiment toward GMOs and ultimately play an important role in combating mis-information.

## 1 Introduction

GMOs are defined as organisms containing altered DNA.^1^ From a historic perspective, large-scale commercial production of GM crops began around 1994 with the introduction of the Flavr Savr tomato and has rapidly expanded globally since.^2^ Specifically, between 1996 and 2014, global GM production has grown from 1.7 million hectares to 182 million hectares with the United States, Brazil, Argentina, Canada, and India representing the majority of the growth.^2^ As an example of market share, in the United States, GM soybeans represent 94% of the overall soybean production, GM cotton 94% of overall cotton production, and GM corn 92% of overall corn production.^3^

In the scientific community, it is well acknowledged that GM crops have substantial benefits for farmers, consumers, and the environment.^4^ From an environmental perspective, GM-based crops such as transgenic Bt corn and Bt cotton utilize less pesticides and facilitate greater abundance of non-target invertebrates than their non-transgenic analogs.^5,6^ From a yield perspective, on average, GM crops have resulted in a 22% increase in agricultural yield and a 68% increase in farmers’ profit.^7^ Interestingly, these profit gains were 60% points higher in developing countries compared to developed ones due to stronger GM yield gains, higher pesticide cost savings, and lower GM seed prices than in developed countries.^7^ From a food security perspective, GM plants can also be designed to grow in sub-optimal conditions where their non-GMO cultivar analogs are unable to flourish.^7^

From a nutritional perspective, GM crops have been shown to enhance food quality and nutrient composition, critical for tackling malnutrition and associated nutritional diseases.^8^ For example, L-1 transgenic corn has a 169-fold increase in beta-carotene, a 6-fold increase in vitamin C, and a 2-fold increase in folate compared to its non-GMO cultivar.^9^ Likewise, “Golden Rice”, which is a GM-based rice, which contains higher concentrations of beta-carotene, has the potential to mitigate some of the 250,000 yearly cases of blindness associated with Vitamin A deficiency.^10^ From an organoleptic and spoilage perspective, GM crops can be modified without sacrificing taste or quality. An example of this is a GM banana crop that increases shelf life by reducing the expression of two transcription factors while still maintaining the quality and taste of the banana.^11^ Furthermore, in a blind tasting of GM and non-GM tomatoes, 60% of the participants preferred the taste of GM tomatoes over their non-GM counterparts.^12^

Despite their benefits, GMOs are met with heavy criticism. Currently, 26 countries including France, Germany, Italy, Mexico, Russia, China, and India (19 of which are in the European Union (EU)) have partially or fully banned GMOs.^13^ Another 60 countries have significant restrictions on GMOs.^14^ One reason for this opposition to GMOs is because of the perceived weak agricultural benefits of GMOs compared to their potential risks.^15^ There is also a noted lack of trust and confidence by the public in regulatory processes behind GMOs.^15^ In a 2015 study regarding consumer perceptions of GMOs, 57.4% of the participants doubted the reliability of studies showing positive health effects of GMOs, and 64.1% viewed GMO media reports as untrustworthy.^16^ As a whole, consumers are also willing to pay 29–45% extra for non-GM products to avoid GM foods.^16^ In a consumer trend survey of more than 2,200 adults in the United States, 46% found the phrase “non-GMO” as more appealing when purchasing food/beverage products, 34% found the phrase neither less nor more appealing, 8% as less appealing, and 12% undecided.^17^

As of September 2021, 4.48 billion people globally use social media. Specifically, in the United States, over 90% of the population uses the internet and 72.3% use social media.^18,19^ Web-crawling is a social media analysis method for gathering and analyzing “big data” from social media and other sites on the internet based on a user-defined query to provide insight into public opinions.^20^ These data can be further analyzed via various natural language processing (NLP) tools for sentiment and emotion analysis.^21^ For their application, sentiment analysis of web-crawled data has become an important tool for understanding many topics such as sentiment toward the COVID-19 vaccine,^22^ sentiment toward political discourse,^23^ and social preferences toward infrastructure.^24^

With a large proportion of the population using social media, utilizing a web crawler allows for a majority of public attitudes related to a specific subject to be measured and analyzed at a more significant scale compared to traditional data collection methods, such as surveys.^25^ As GM technology continues to grow technically but continues to be controversial in different populations, developing tools to understand public sentiment associated with GMOs will be integral toward understanding issues of adoption over the coming decades. In this research, we present an analysis of sentiment toward GMOs across multiple social media and web-based platforms.

## 2 Materials and Methods

### 2.1 Data Collection

Social media– and web-based articles (content on websites such as forums, news, blogs, reviews, images, and videos) in English related to GMOs using a consumer insight tool called Brandwatch^26^ were collected between May 1, 2019, and May 31, 2021. Specifically, the commercial software tool employs web crawlers to first search through social media and web pages on the provided search index terms in a query.^27^ To scrape Twitter, the software creates channels to monitor specific query-related accounts.^26^ For other sources, it collects full data fire hoses (streams of data from a digital source in real-time) from social media sites Reddit and Tumblr and full data fire hoses from web pages of blogs, forums, review sites, social networks, news outlets, and video sites.^26^ The software also collects specific Asia-Pacific regional sources such as QQ, Naver, Baidu, Daum, Qzone, China Daily, Sohu, Tistory, FC2, and thousands of APAC sites.^26^

The query we used searched for related mentions in English from May 1, 2019, to May 31, 2021.

The Boolean string used initially in the search: (GMO OR GMOS OR “genetically modified food” OR “genetically modified foods” OR gmo OR gmos OR #GMO OR #GMOS OR #geneticallymodifiedfoods OR #geneticallymodifiedfood)

The Boolean operator “OR” searches for at least one mention of the listed terms separated by “OR”. The Boolean operator “AND” searches for mentions containing all the listed terms separated by “AND”. The Boolean operator “NOT” searches for mentions containing terms that come before the “NOT” that do not include the terms that come after the “NOT.” In this query, the software searches for mentions containing a reference to at least one of the terms in the query. The hashtags were used to include GMO-related categorizations on social media such as Twitter for mentions that do not explicitly mention GMOs but still refer to GMOs.

The initial Boolean string results showed multiple irrelevant mentions to vaccines (mostly related to the COVID vaccine) and genetically modified mosquitoes in Florida. Therefore, in order to eliminate these “false positives,” the following Boolean term was added NOT (vaccine OR mosquitos OR florida)

Therefore, the final query used in search for related mentions in English from May 1, 2019, to May 31, 2021:

(GMO OR GMOS OR “genetically modified food” OR “genetically modified foods” OR gmo OR gmos OR #GMO OR #GMOS OR #geneticallymodifiedfoods OR #geneticallymodifiedfood) NOT (vaccine OR mosquitos OR florida)

### 2.2 Data Analysis

The software analyzes the data, categorizing the mentions on source, country, sentiment(s), and emotion exhibited. The software employs natural language processing to model linguistic features that indicate sentiment and emotion using the context the data is provided in.^21^ It categorizes overall sentiment as positive, negative, or neutral for each post. The emotional analysis algorithm categorizes the posts as displaying “disgust,” “joy,” “sadness,” “anger,” “fear,” or “surprise.”^21^

## 3 Results

**Figure 1.**
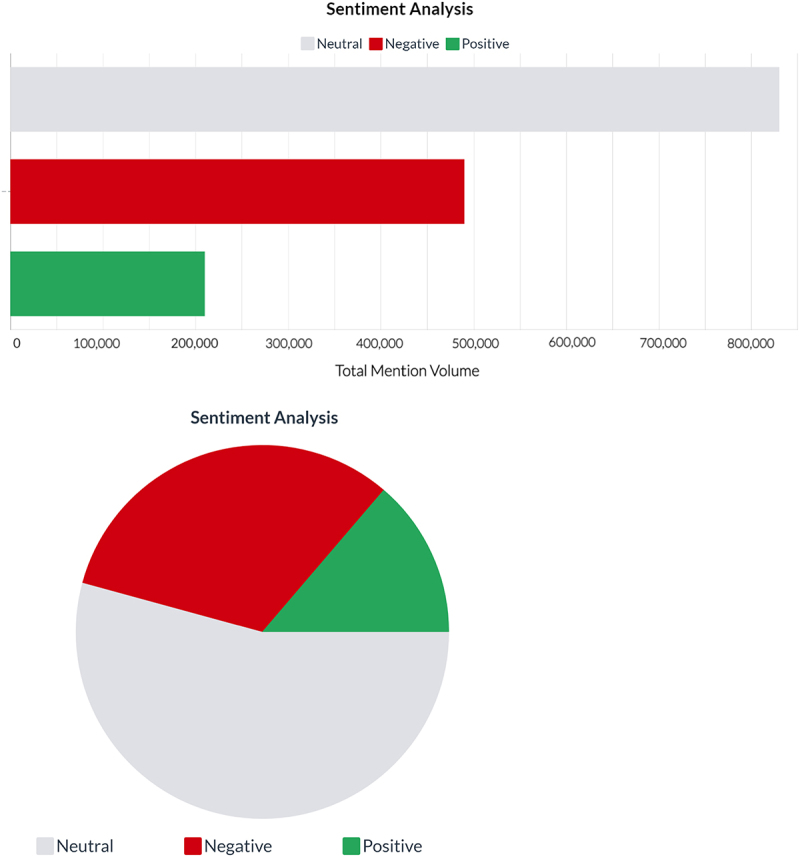
Sentiment analysis displaying the categorization of the mentions as having expressed neutral, negative, or positive sentiment. Figure extracted from Brandwatch software.

**Figure 2.**
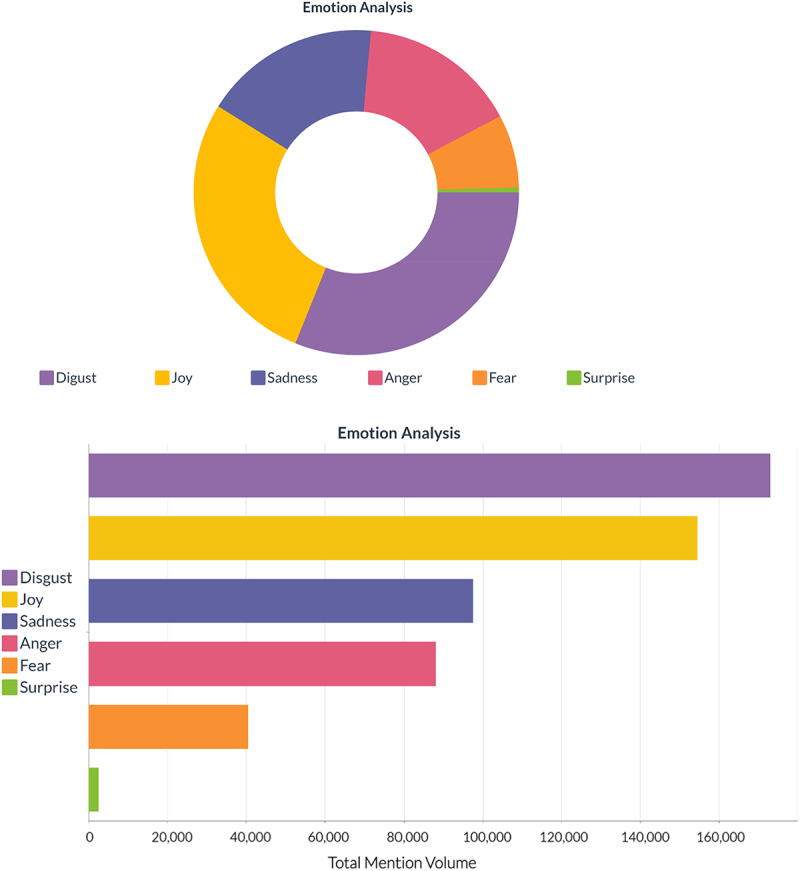
Emotion analysis displaying the categorization of mentions as having expressed disgust, joy, anger, fear, or surprise. Figure extracted from Brandwatch software.

From May 1, 2019 – May 31, 2021, the public sentiment regarding GMOs with respect to the provided query resulted in a collection of over 2 million English mentions from 553,000 unique authors. Out of the 2 million mentions, 54% (1,080,000) were categorized as having a neutral sentiment, 32% (640,000) as having a negative sentiment and 14% (280,000) as having a positive sentiment (Fig. 1). With respect to the emotion analysis, 31% of the mentions were categorized as having expressed disgust, 28% expressed joy, 18% expressed sadness, 16% expressed anger, 7% expressed fear, and 1% expressed surprise (Fig. 2).

**Figure 3.**
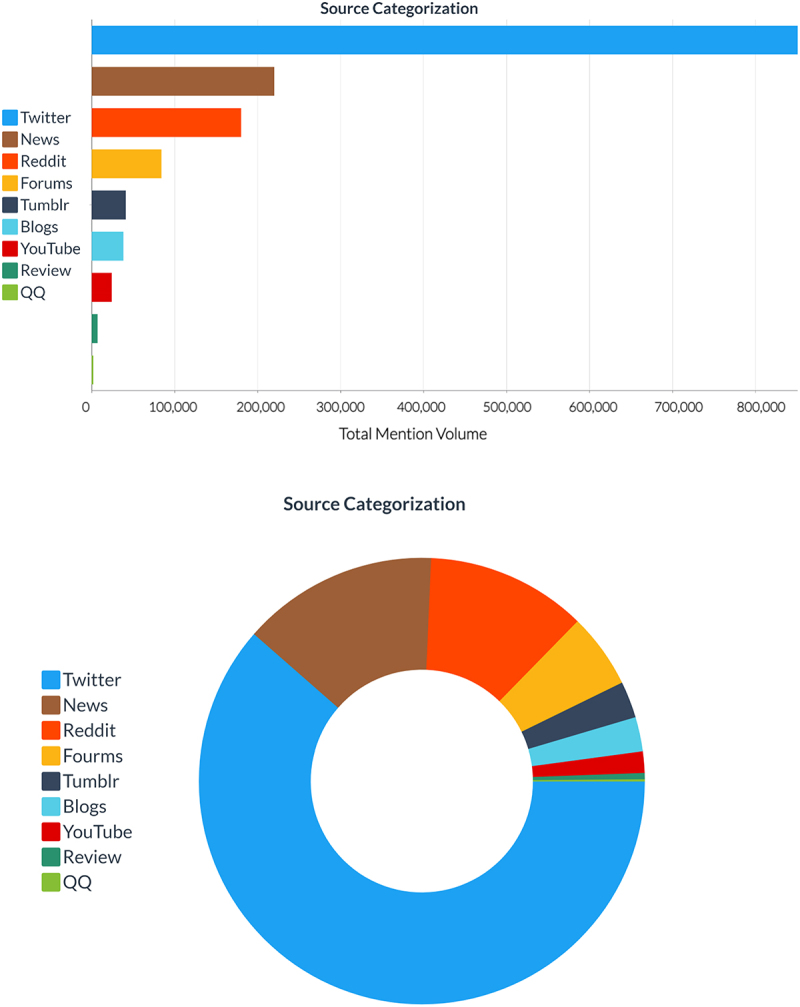
Categorization of mentions related to the GMO defined query. Figure extracted from Brandwatch software.

Social media was the largest category of mentions (85%) with 62% of the total mentions coming from Twitter, followed by Reddit (12%), forums (6%), blogs (2%), Tumblr (2%), YouTube (1%), and QQ (1% (rounded to 0%)) (Fig. 3). In total, approximately 85% of the mentions were collected from “social media,” and the remaining 14% were crawled from “news” from local and national web sources.

**Figure 4.**
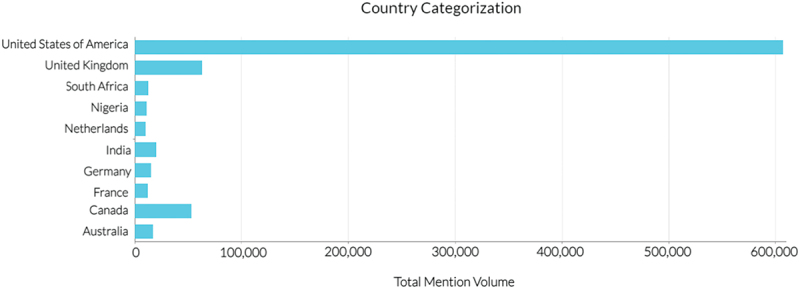
Volume of mentions from the top 10 countries. Figure extracted from Brandwatch software.

Among English-speaking countries, the United States was the largest source of data with over 600,000 mentions. The United States had significantly more mentions than those of the UK and Canada combined (Fig. 4).

## 4 Discussion

### 4.1 Application

The results indicate that the major sentiments toward GMOs were neutral (54%) or negative (32%) (Fig. 1) and were expressed on Twitter (62%) (Fig. 3). The results also indicated that the major emotion toward GMOs was disgust (31%). The combination of unpleasant emotions (e.g. disgust, sadness, anger, and fear) accounted for 71% of the total emotions expressed (Fig. 2). With respect to social media, it is important to understand that negative sentiment has a greater reach than positive sentiment, particularly on Twitter where tweets with negative sentiment are more likely to become viral.^28^ On average, each positive word in a tweet results in a 7.14% decrease in the number of retweets, whereas each negative sentiment word results in an average 3.46% increase in the number of retweets.^28^ Consequently, the greater reach associated with negative sentiment suggests that the impact of sentiment should not solely be measured in absolute numbers due to the apparent multiplier effect of negative sentiment. From a policy perspective, negative public sentiment and emotions toward GMOs (even in relatively small absolute numbers) on social media has the potential to disproportionately affect various stakeholders including decision makers (i.e. regulators, elected politicians) and companies (i.e. marketing and future technology adoption) who may be less keen to develop and commercialize GMO-based technologies based on negative sentiment and a lack of confidence in marketability.

As the impact of social media on policy and commercialization becomes greater,^29^ approaches to better educate the general public about GMOs via social media will be critical toward facilitating rational science-based discussions. Based on this study, an initial focus on Twitter is warranted based on Twitter having the highest absolute number of mentions of all the different platforms (Fig. 3). Approaches including content moderation (i.e. a mechanism to identify and remove or otherwise address inaccurate information) and the use of social media bots (i.e. autonomous bot that operates on social media) that identify and address misinformation by posting a reply with the correct scientific information exist^30^ but generate controversies^31^ and challenges of their own.^32^ While social media is considered the “new battle-ground” for these types of discussions, traditional education, including the integration of GMOs in the standard curriculum should also be considered as a long-term approach toward understanding GMOs as a technology. For example, short-term environmental education has been shown to help increase student’s consideration and motivation to preserve nature.^33^ Based on the results of the short-term education study, integration of GMOs in standard curriculum could have significant impact especially in the United States (which had the most GMO-related discussion among English-speaking countries) to shift sentiment on GMOs (Fig. 4).

### 4.2 Limitations

Due to the large nature of the data-set (*n* > 2 M), we were unable to download such a large data set to process the mentions through a filtering program to prevent false positives (non-GMO related mentions) from being included in the dataset. To reduce the number of false positives, in the initial Boolean search query, additional terms were excluded based on the initial results. For example, “NOT (vaccine OR mosquitos OR Florida)” was added to prevent the high number of topics related to vaccines and mosquitos in Florida that were largely unrelated to GMOs. Additional data filtering using various tools including Natural Language Processing (NLP) could be used in future studies to further refine the results to include various forms of sentiment analysis, opinion mining, and social network analysis (SNA).^34^ While the commercial software used for this study has its own NLP, the methods and code associated with the commercially used NLP were not provided by the company and therefore it is unknown to what degree the software correctly differentiated between a positive sentiment of a non-GMO related mention and that of a GMO-related mention. In one study, the commercial software’s analysis of a brand’s advertisement showed substandard results compared to the standard human content analysis.^35^ Specifically, the commercial software showed unreliable brand identification and sentiment polarity compared to the standard human analysis.^35^

Future analysis using custom web-scraping tools for academic purposes linked to open-source NLP tools will be essential for further insights. Further analysis of social media via various natural language processing (NLP) tools would allow a more in-depth understanding of specific issues the public has with GMOs, which could allow for a more targeted science-based response. For instance, an NLP-based sentiment analysis using ensemble methods outperforms traditional bag-of-words approach by 3–5%.^35^

The query was conducted in English thereby not including potentially influential non-English mentions, especially for large non-primary English-speaking countries that use Twitter such as Japan, India, Brazil, and Indonesia, which is a significant limitation.^36^ Furthermore, public sentiment specific to countries was not collected. For example, while the United States accounted for 600,000 mentions, or 30% of the total 2 million mentions, not all the mentions’ locations were identified. Specifically, the categorization is based only on the mentions whose source location was identified. Consequently, while the United Sates had a disproportionate influence on this data set, having country-level sentiment data would offer further insights at a national level.

## 4.3 Conclusions

When considering the above-mentioned study limitations, the overall results suggest a significant problem with continued adoption of GMOs in the public. Specifically, while the potential for GMOs to address multiple aspects of human nutrition,^8–10^ food security,^7^ and agricultural adaptation to climate change^7^ exists, the results suggest that adoption of GMO-based technologies in these areas may be challenging due to the negative public perceptions toward GMOs identified in this study and others.^4^ While GMO-based technologies such as CRISPR-cas9 continue to develop both scientifically and commercially, it will be equally critical to have parallel efforts focused on communicating accurate information to the public in order to ensure the viability of GMOs as a tool to address current and projected challenges in food production including projected 2050 population growth estimates, food security, and climate change.
